# Paths to stability – actin regulation of adherens junction mechanics

**DOI:** 10.1242/jcs.264055

**Published:** 2025-11-19

**Authors:** John James, Lucinda B. A. Winn, Peter Mottram-Epson, Darius Köster

**Affiliations:** Warwick Medical School, Biomedical Sciences and Centre for Mechanochemical Cell Biology, CV4 7AL Coventry, UK

**Keywords:** Cadherin, Cell–cell adhesion, Adherens junction, Actin cytoskeleton

## Abstract

The ability of cells to stick to each other and form tissues is mediated by protein complexes at the plasma membrane, such as adherens junctions (AJs). Key aspects of AJ stability are the biomechanical properties of the constituent proteins and the forces generated by the associated actin cytoskeleton. This Review concisely overviews our current understanding of how these factors play out at different length scales. When actomyosin pulls on the cadherin–catenin complex, the molecular interactions within the complex lead to an increase in AJ stability. Transcellular E-cadherin clusters are dynamically maintained by constant turnover and recruitment of actin-binding proteins organises the internal actin cytoskeleton. Among these are actin polymerisers that sustain the actin network and provide the mechanical forces important for AJ integrity. Finally, the distribution of AJs around the cell periphery and the long-range organisation of the associated actin bundles could contribute to maintaining AJ stability across tissues. We conclude with a summary of recently developed biophysical tools useful for the study of AJ mechanics and a few open questions that we expect to see answered in the not-too-distant future.

## Teaser

**Summary:** A Review of E-cadherin-mediated cell-cell adhesion, focusing on recent advances in our understanding how actin cortex dynamics affect adherens junction stability.

## Introduction

**Fig. 1.**
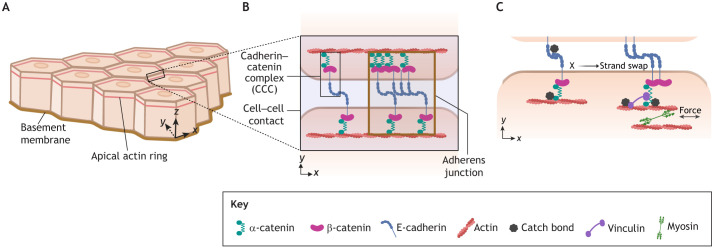
**AJs maintain tissue integrity by maturing in response to actomyosin generated tension at cell–cell contacts.** (A) AJs are formed in the apical region of polarised epithelial tissues. Subsequent figures show a top-down view of an *x*-*y* plane at the apical region. (B) Schematic defining the key components involved in cell–cell adhesion, as discussed in this Review. Cadherin–catenin complexes (CCCs) hold together the actin cytoskeletons of adjacent cells at regions of cell–cell contact. Clustering of CCCs forms AJs. (C) Pulling forces exerted on CCCs changes their bio-mechanical properties. The E-cadherin trans interaction changes from an X- (left) to a strand-swap (right) conformation and α-catenin recruits vinculin, which strengthens the binding of α-catenin to F-actin. Florette indicate catch bonds, which increase in lifetime with applied force (E-cadherin X dimer, α-catenin tail domain–actin and vinculin tail domain–actin). Created in BioRender by Winn, L., 2025. https://BioRender.com/l1sc933. This figure was sublicensed under CC-BY 4.0 terms.

Adherens junctions (AJs) link adjacent cells and connect their actin cytoskeletons. AJs are mechanosensitive structures, with actin-based contractility contributing to their maturation and enhancing adhesion between cells at contact sites. AJs typically form in a region known as the zonula adherens, located slightly below the apical pole in a polarised epithelium (Mangeol et al., 2024), and are therefore best studied in epithelial cells (Fig. 1A). This region is enriched in bundles of actin filaments, which provide the mechanical forces required to maintain cell–cell contacts. The traditional view suggests that these contractile bundles, which run parallel to the surface of cell–cell contact, straighten out the membrane, thereby keeping cells in contact with each other (Cavey and Lecuit, 2009). However, recent studies point to a more complex picture, with multiple mechanochemical pathways coming together to stabilise both the AJs and the underlying actin cytoskeleton.

In this Review, we present four distinct paths that contribute to the stability of AJs. These paths function on different length scales, beginning with molecular interactions within the cadherin–catenin complex (CCC) – the structural unit of an AJ – where α-catenin binding is strengthened by pulling forces generated by the actomyosin machinery. Next, clusters of CCCs come together and are dynamically maintained by E-cadherin turnover, actin network contractility and the recruitment of actin-binding proteins. Large-scale interdigitations between adjacent cells originate from actin polymerisation at the plasma membrane, generating pushing forces to hold membranes together. Finally, at a cellular scale, the distribution of AJs around the cell periphery impacts the overall organisation of the actin cortex and potentially how forces are distributed across tissues. For readers interested in entering the field, we summarise a few recently developed biophysical methods that have been crucial for many of these findings and have the potential to deliver interesting insights in the future.

## The CCC and actomyosin contractility

First, we discuss how the bio-mechanical properties of molecular interactions between key CCC components, together with mechanical forces generated by actomyosin, enhance the stability of AJs. In epithelial cells, the CCC consists of epithelial (E-)cadherin (encoded by *CDH1*), α-catenin (encoded by *CTNNA1*) and β-catenin (encoded by *CTNNB1*) (Fig. 1B). The extracellular domain of E-cadherin, a transmembrane protein, binds to the extracellular domain of another E-cadherin in a neighbouring cell (trans interaction) (Harrison et al., 2010). The intracellular domain of E-cadherin interacts with several cytoplasmic proteins, most importantly β-catenin and α-catenin. Catenins are a diverse group of proteins that stabilise cell adhesion complexes upon binding to them and can play important roles in the nucleus to regulate gene expression when dissociated from them (McCrea and Gottardi, 2016; McCrea and Gu, 2010). Although β-catenin binds to both the E-cadherin juxta-membrane region (Huber and Weis, 2001) and the α-catenin head domain (Pokutta and Weis, 2000), a crystal structure of the complex has proved difficult to isolate. The presence of several flexible linker regions in the constituent proteins leads to an ensemble of conformations, the stabilisation of which requires an external force (Bush et al., 2019; Farago et al., 2021). This force is provided by actomyosin contractility – the α-catenin tail domain in turn binds to actin filaments (Rimm et al., 1995), linking the CCC to the actin cytoskeleton. This mechanical coupling of adjacent actomyosin networks through the CCC is crucial not only for the stability of CCCs but also for transmitting the forces generated by actin polymerisation and myosin-dependent contraction (Mège and Ishiyama, 2017; Ratheesh and Yap, 2012).

On a mechano-molecular level, CCCs are composed of a series of catch bonds – molecular hooks whose bond lifetime increases with the pulling force applied to them (within a given force range) (Hertig and Vogel, 2012) (Fig. 1C). E-cadherins from adjacent cells bind in a transient ‘X’ conformation that functions as a catch bond (Rakshit et al., 2012) and can undergo a transition to a stronger strand-swap-dimers conformation (Li et al., 2013; Maker et al., 2022). Next, α-catenin exhibits multiple force-responsive conformational changes that strengthen AJs (Noordstra et al., 2023b). First, structural studies have revealed that force induces a conformational change within the α-catenin tail domain that enhances actin binding (Ishiyama et al., 2018). This α-catenin-actin bond is a biphasic molecular slip-catch bond, which has a 20-fold increase in bond lifetime to 1.2 s at 10 pN (catch-bond regime) but displays decreasing bond lifetimes with increasing force at forces higher than 10 pN (slip-bond regime), as measured *in vitro* using optical traps (Buckley et al., 2014). Second, binding of multiple CCCs to filamentous actin, together with a conformational change in α-catenin induced by β-catenin, further strengthens the catch bond, leading to a transition from fluid (wherein actin can move along CCCs) to solid (wherein actin movement is stalled with respect to CCCs) behaviour under force (Arbore et al., 2022). Third, contractile forces from the actomyosin network stretch α-catenin, revealing a cryptic vinculin-binding site between the α-catenin head and tail domains (Yao et al., 2014; Ishiyama et al., 2013). Vinculin then binds simultaneously to α-catenin and actin filaments to reinforce the link between CCCs and the actin cytoskeleton. The vinculin–actin bond also acts as a directional catch bond with a 12 s lifetime at 8 pN when forces are applied towards the actin pointed (−) end, whereas force applied in the opposite direction leads to a slip-bond behaviour (Huang et al., 2017). Interestingly, vinculin and α-catenin are structurally and evolutionarily closely related proteins and a recent study has shown that in the absence of α-catenin, vinculin can bind directly to β-catenin, potentially to act as a fail-safe and maintain CCC structure (Hazan et al., 1997; Morales-Camilo et al., 2024). Thus, the CCC is a protein complex that connects E-cadherins of adjacent cells with the actin cytoskeleton, and multiple molecular interactions within the CCC are strengthened by contractile forces generated by the actomyosin machinery in both cells.

## CCC clustering and dynamic maintenance of AJs

The second pathway to AJ stabilisation involves the ability of CCCs to come together and form larger arrays along the plasma membrane. This effect is largely driven and cooperatively enhanced by interactions between lateral neighbour E-cadherins (cis interactions) within the same plasma membrane of the same cell and interactions between opposite neighbour proteins (trans interactions) between adjacent cells. Cis interactions are essential for the formation of AJs (Harrison et al., 2011) and mutations that selectively prevent either cis or trans interactions increase the average dissociation rate of E-cadherin from clusters (Thompson et al., 2021). *In vitro* experiments using minimal membrane systems have shown that the extracellular domain of E-cadherin alone can give rise to arrays of cis-trans clusters more than 100 nm long when tethered to giant unilamellar vesicles (Harrison et al., 2011)*.* In cells, expression of the E-cadherin extracellular domain alone led to formation of unstable clusters; however, expression of chimeric proteins combining the extracellular domain of E-cadherin with the actin-binding tail domain of α-catenin showed that actomyosin-generated forces play an important role in formation and maintenance of E-cadherin clusters (Hong et al., 2013). Increasing actin contractility correlates with a local increase in cluster intensity, suggesting an inside-out signalling mechanism (Engl et al., 2014). *In vitro*, α-catenin binding to actin induces a force-dependent conformational change in the α-catenin actin-binding domain, which promotes dimerisation (Ishiyama et al., 2018). Afadin, a key substrate in the Ras-phosphoinositide 3-kinase (PI3K)-AKT pathway, has been shown to promote this dimerisation synergistically with vinculin (Gong et al., 2025). Importantly, α-catenin oligomerisation, which has now been observed in myosin-1c-enriched cell cortices (Troyanovsky et al., 2025), is emerging with increasing evidence as a key mechanism for inside-out clustering. Furthermore, strengthening of the connection between AJ and actin filaments by vinculin, together with myosin contractility, promotes the switch from the X to the strand-swap conformation of the E-cadherin extracellular domains (Koirala et al., 2021), decreasing the average dissociation rate of E-cadherin from clusters (Thompson et al., 2021).

The stability of the AJs depends not only on the strength of individual CCCs but also on the dynamic maintenance of the CCC clusters by constant turnover of their components. Two-colour fluorescence recovery after photobleaching (FRAP) experiments on AJs between cells either expressing E-cadherin–GFP or E-cadherin–DsRed has revealed that turnover rates of E-cadherin are similar between pairs of AJs, but they can vary over an order of magnitude along the cell periphery (*K*_off_=10^−3^ s^−1^, 10^−2^ s^−1^; meaning that it takes between 1000 and 100 s for a single E-cadherin to leave the site of photobleaching). This indicates that turnover rates are controlled very locally, within a few micrometres (de Beco et al., 2015). Most interestingly, the same study reported an approximately twofold increase of E-cadherin turnover in the presence of local stress applied perpendicularly to the AJs, which indicates that increased influx of E-cadherins into the adhesion is coordinated with increased endocytosis (de Beco et al., 2015). These turnover rates are still very slow compared to the time scales of molecular interactions but indicate that withstanding increased stress requires a quicker replenishment of E-cadherin molecules. Thus, although the stability of AJs is commonly linked solely to the maturation of CCCs upon mechanical stress, the ability to replenish CCC components is also essential to maintain AJ stability.

**Fig. 2.**
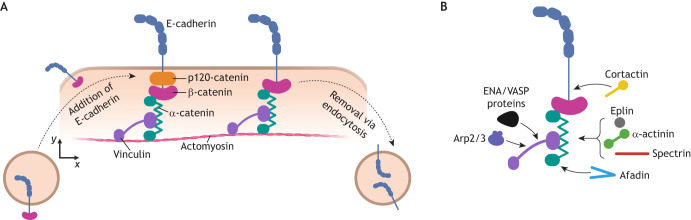
**Dynamic maintenance of AJs.** (A) E-cadherins are continuously replenished at existing AJs. New E-cadherins arrive at AJs via intracellular vesicles and membrane flows. The CCC at AJs is stabilised by the presence of p120-catenin. When p120-catenin is dissociated from the CCC, E-cadherins are removed by endocytosis for degradation or recycling. (B) Schematic representation of the actin binding proteins that are recruited by known binding partners within CCCs. These proteins, which include actin nucleating, elongation and crosslinking factors, dynamically regulate and maintain the actin cytoskeleton associated with cell–cell contacts. Created in BioRender by Winn, L., 2025. https://BioRender.com/vctua08. This figure was sublicensed under CC-BY 4.0 terms.

p120 catenin (encoded by *CTNND1*) is responsible for retaining E-cadherin at the plasma membrane by binding its juxta-membrane domain and is also important for E-cadherin clustering (Yap et al., 1998). Consequently, the recruitment of p120-catenin to AJs increases when tissues are under mechanical stress (Iyer et al., 2019). Dissociation of p120-catenin from E-cadherin leads to endocytosis (i.e. internalisation) of E-cadherins for degradation or recycling (Kowalczyk and Nanes, 2012) (Fig. 2A). Regulation of p120-catenin localisation is thought to be controlled by the Src kinase pathway, which is centrally important in epithelial cells for regulating proliferation, adhesion and migration (Ortiz et al., 2021). Although it has been proposed that phosphorylation of p120-catenin downstream of the Src kinase regulatory pathway dissociates its interaction with E-cadherin, the molecular interactions involved and how they are tuned by mechanical cues remain incompletely understood (Mendonsa et al., 2020; Reynolds, 2010).

New E-cadherins arrive at AJs by fusion of intracellular vesicles and through plasma membrane flows. An interesting aspect of this process is that clustering of E-cadherin creates a diffusion trap (i.e. reduces the mobility of free E-cadherins), which, in turn, promotes the growth of clusters (Chandran et al., 2021). Computational models suggest that as clusters grow, the contact angle between cells increases, leading to an increase in membrane tension, which further leads to immobilisation of E-cadherins and strengthening of CCCs (Chen et al., 2021).

Dynamic regulation of the actin cytoskeleton is also crucial for AJ stability. Actin associated with AJs is remodelled by the recruitment of several actin-binding proteins and forms a band of parallel actin filaments around the cell circumference (Fig. 2B). This actin band is under tension generated by RhoA-regulated myosin contractility, which is activated after cell attachment (Arslan et al., 2024; Yu-Kemp et al., 2021). In addition to its structural role within CCCs, α-catenin recruits actin crosslinkers, namely the α-actinin family (Imamura et al., 1999) and eplin (also known as LIMA1) (Abe and Takeichi, 2008), which aid in formation and maintenance of the compact band of actin filaments. Actin filament organisation is also influenced by the recruitment of vinculin, which can not only bind to further actin filaments but also bundle them via dimerisation of its tail domains (Jannie et al., 2015). Spectrin, a cytoskeletal protein best known for its role in red blood cell mechanics, is also recruited by α-catenin and helps form ordered actin filament networks (Machnicka et al., 2014). These dense actin networks formed by CCCs are likely to contribute to a ‘picket fence’ environment, limiting E-cadherin diffusion (Sako et al., 1998). Vinculin also binds the actin nucleation factor actin-related protein 2/3 complex (Arp2/3) and the actin elongation factors M-Ena (also known as ENAH) and VASP (Brindle et al., 1996; DeMali et al., 2002), which might contribute to sustaining these actin networks, although their interaction with vinculin is not essential for their recruitment to AJs (James et al., 2025; Oldenburg et al., 2015).

Taken together, these studies indicate that the clustering of the CCC, the core unit of AJs, constitutes an important element in the stabilisation of AJs and control of their dynamics. The accumulation of CCCs is fostered by a contractile actomyosin network and, in turn, acts as a scaffold for the recruitment of many other regulatory and actin-binding proteins that help to shape and maintain actomyosin network organisation.

## Actin polymerisation at AJs

The third pathway to AJ stability involves the role of actin polymerisation in reinforcing and remodelling cell–cell contacts. The process of actin polymerisation is controlled by the localisation and regulation of actin-polymerising proteins. Briefly, polymerisation is initiated by actin nucleating factors – primarily, formins for linear polymerisation and Arp2/3 for branched polymerisation. Existing linear filaments are extended by elongation factors that include ENA and VASP homology proteins (hereafter ENA/VASP proteins) and some formins, whereas branched polymerisation can be promoted by nucleation-promoting factors, like the WAVE complex, that activate Arp2/3 (Pollard, 2016; Molinie and Gautreau, 2018). Although actin polymerisation has been shown to occur at AJs (Vasioukhin et al., 2000), its function has yet to be fully understood. Initial reports suggested that actin polymerisation is important for the formation of AJs, whereas contractility maintains their stability. However, it is becoming increasingly clear that there is an equilibrium between these processes, with branched actin polymerisation at AJs found to occur even in mature monolayers. Both linear and branched actin-nucleating factors, including formin 1 (recruited by α-catenin), mDia1, mDia2 and Arp2/3 (Kobielak et al., 2004; Carramusa et al., 2007; Acharya et al., 2017; Han et al., 2014), have been found at AJs, along with elongation and nucleation-promoting factors, including ENA/VASP proteins, WAVE2 (also known as WASF2) and N-WASP (also known as WASL) (Oldenburg et al., 2015; Lee et al., 2016). Other positive regulators of actin polymerisation, such as CDC42, RAC1, IQGAP1 and cortactin, have also been reported in AJs (Braga et al., 1997; Noritake et al., 2004; Han et al., 2014).

**Fig. 3.**
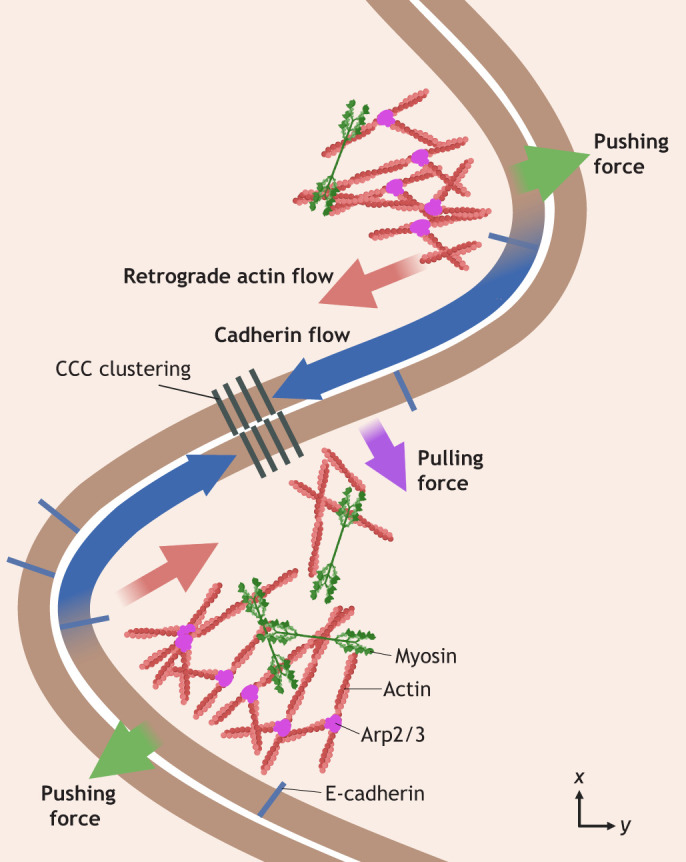
**Schematic depicting forces and protein flows generated by branched actin polymerisation at AJs.** Actin polymerisation at the cell membrane generates pushing forces that create a protrusion into the adjacent cell. Retrograde flow of actin from the tip of the protrusion to its base is driven by actin polymerisation at the tip and actomyosin contractility at the base. The binding of actin to CCCs couples the retrograde actin flow to E-cadherin flows in the membrane leading to E-cadherin clustering. Pulling forces generated by retrograde actin flow and associated actomyosin contractility thus contribute to maturation of CCCs. Created in BioRender by Winn, L., 2025. https://BioRender.com/cx1dvgx. This figure was sublicensed under CC-BY 4.0 terms.

Branched actin polymerisation is accompanied by an increase in the stability of the cell–cell contact. The recruitment of Arp2/3 correlates with increased cell junction stability (James et al., 2025) and treating cells with the Arp2/3 inhibitor CK666 leads to gaps in AJs (Senju et al., 2023). Cortactin, a cytoplasmic protein that connects several signalling pathways with actin cytoskeleton restructuring (Schnoor et al., 2018), binds E-cadherin and is recruited to AJs (Han et al., 2014). Within an actin branch, cortactin stabilises Arp2/3 (Liu et al., 2024) and releases the Arp2/3 activator WASP (also known as WAS) to promote further polymerisation (Fregoso et al., 2023). Knockout of cortactin in myocardial endothelial cell monolayers results in junction fragmentation, changes in actin filament distribution and reduced barrier function (Moztarzadeh et al., 2024). Branched actin polymerisation generates cell membrane protrusions that extend into adjacent cells (Senju et al., 2023) and might support adhesion in multiple ways. For example, exponential expansion of branched networks through cooperative branched and linear actin polymerisation could generate pushing forces at the membrane, helping to keep cell membranes together (Li et al., 2021). Additionally, a molecular clutch coupling actin retrograde flow to E-cadherins in the membrane could contribute to CCC clustering (Noordstra et al., 2023a). Retrograde flow driven by actin polymerisation and actomyosin contractility creates a pulling force contributing to maturation of CCCs (Zhitnyak et al., 2020; Noordstra et al., 2023a). Furthermore, an increase in the surface area of the cell–cell contact could promote the formation of more CCC clusters, thereby increasing adhesion (Fig. 3).

In summary, actin polymerisation, especially the formation of branched actin networks by Arp2/3, plays an active and multifaceted role in stabilising AJs, ranging from providing a substrate for the binding of CCCs and contractile force generation by myosin motors, to polymerisation forces physically pushing adjacent cell membranes together.

## AJ distribution and actin architecture

**Fig. 4.**
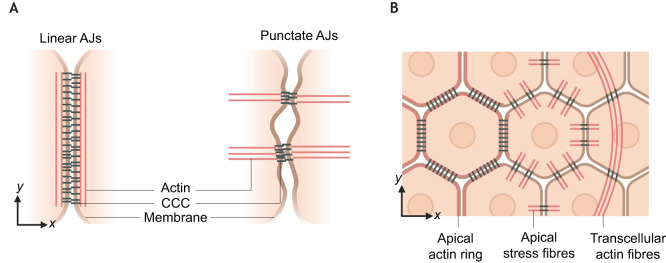
**AJ distribution and actin architecture can vary at a cellular scale.** (A) Two main forms of AJ organisation are observed in cell layers – linear AJs, in which actin bundles are organised parallel to the surface of cell–cell contact, and punctate AJs, in which actin bundles are organised perpendicular to the surface of cell–cell contact (Angulo-Urarte et al., 2020). (B) The traditional view of actin arrangement in epithelia is an apical actin ring associated with linear AJs around the cell. However, several recent studies have shown the presence of apical ‘stress fibres’, which can be coordinated across punctate AJs to form transcellular actin fibres (TAFs) that span multiple cells. Created in BioRender by Winn, L., 2025. https://BioRender.com/ew9bx0h. This figure was sublicensed under CC-BY 4.0 terms.

The fourth path to stability concerns the spatial organisation of AJs across the cell and the corresponding architecture of the actin cytoskeleton. The spatial distribution of AJs along a cel–cell contact, together with the architecture of the underlying actin network, can vary. Commonly, cells display so-called ‘linear’ AJs, where actin bundles run parallel to the surface of the cell–cell contact, with AJs distributed uniformly along the surface of contact (Fig. 4A). However, cells can also exhibit ‘focal’ or ‘punctate’ AJs, which appear as spikes that are dotted along the cell–cell contact, with actin bundles arranged perpendicular to the surface of contact (Malinova and Huveneers, 2018). We will here call these ‘punctate adherens junctions’ (pAJs) to avoid confusion with the cell–substrate adhesions known as focal adhesions. pAJs are commonly seen in endothelial cells but have also been reported to be formed at the apical surface of epithelial monolayers as well as during epithelial-mesenchymal transition (Yonemura et al., 1995; Zhitnyak et al., 2020). Actin bundles emanating from pAJs can stretch across the cell and connect to pAJs on the other side, resembling stress fibres that connect integrin-based focal adhesions between the cell and a non-cell substrate (López-Gay et al., 2020). These bundles appear to be coordinated across cell membranes (Ando-Akatsuka et al., 1999), forming structures we will refer to as transcellular actin fibres (TAFs) (Fig. 4B).

The molecular mechanisms that give rise to pAJs and TAFs, as well as their functions in tissues, remain unclear. TAFs have been reported in epithelial tissues such as the *Drosophila* trachea, where they contribute to tissue shortening, to the actomyosin ‘purse string’ formed during wound healing and at tumour boundaries *in vivo* (Begnaud et al., 2016; Mudhar et al., 2018; Pradhan et al., 2023). These contexts suggest a role for TAFs in controlling collective cell migration and maintaining tissue integrity.

The constituent proteins of pAJs are similar to those found at linear AJs and include actin polymerisers, such as ENA/VASP proteins and Arp2/3, as well as scaffolding molecules, such as vinculin and cortactin (Indra et al., 2020). In the *Drosophila* trachea, downregulation of actin contractility, mediated by Rho1, Rok and the myosin regulatory light chain Spaghetti Squash (Sqh), as well as actin polymerisation mediated by Cdc42, Arp2/3 and WASP, leads to a loss of these structures, indicating that formation of TAFs requires a tight balance between actin polymerisation and contractility (Pradhan et al., 2023). Indeed, perturbing contractility by knocking down eplin, an actin-crosslinking protein recruited by α-catenin, leads to the loss of the apical actin ring and the formation of pAJs and TAFs (Abe and Takeichi, 2008). One of the few characteristics that distinguish pAJs from linear AJs is their association with high membrane curvature. This curvature is sensed by members of the BAR family of proteins, which can regulate actomyosin architecture and E-cadherin trafficking (Malinova and Huveneers, 2018). However, it remains unclear whether the high curvature of the membrane promotes the formation of pAJs or whether it is induced by pAJs and their associated actin filaments.

In summary, the spatial distribution of AJs is an important factor in the stability and function of cell–cell adhesion. While linear AJs are commonly formed in epithelial monolayers, providing a tight boundary between cells, the formation of pAJs and TAFs represents an important alternative AJ configuration that leads to increased interdigitation of cell surfaces and strengthened mechanical connections across multiple cells to overcome particular kinds of mechanical constraints; for example, those encountered during collective cell migration during wound healing.

## Biophysical tools to probe the stability of AJs

As outlined above, AJ formation, regulation and organisation are tightly linked to the mechanical forces acting on them and, in turn, they impart mechanical stability to cell monolayers and tissues. Methods to physically manipulate reconstituted protein complexes *in vitro*, in cells and in tissues on different lengths scales have played an important role in the understanding of the biophysical factors relevant for AJs. For example, optical tweezers have proved to be essential in deciphering the catch bond behaviour of AJ components, such as E-cadherin, α-catenin and vinculin (Li et al., 2013; Buckley et al., 2014; Huang et al., 2017). In these studies, the typical assay consists of two optical tweezers each holding a micron-sized bead coated with an actin-binding protein. Passing a single actin filament along the bead allows measurement of the actin-binding time as a function of the applied force, which can be controlled in the pN range (Bustamante et al., 2021). In the past decade or so, the development of new tools to mechanically manipulate cells, to measure forces with high spatial and temporal resolution and to regulate protein activity with high precision using light has flourished. In the following section, we highlight techniques that we deem promising in the study of AJs or cell–cell junctions in general.

Although optical tweezers allow us to measure the force-dependent binding behaviour of individual AJ components *in vitro*, it is very difficult to measure the forces generated at individual adhesion sites in cells or in tissues. Molecular tension sensors based on the principle of Förster resonance energy transfer (FRET) between fluorophores, together with high-resolution fluorescence microscopy, has provided unique insights into the distribution of forces at cell adhesion sites. FRET describes the transfer of energy between an excited fluorophore (called the donor) to another fluorophore (the acceptor, usually of a higher wavelength) when they are in proximity (a few nanometres). The efficacy of this energy transfer is strongly dependent on the distance between donor and acceptor, and each donor–acceptor pair is characterised by the Förster radius beyond which FRET efficiency falls under 50%. A typical tension sensor module comprises a donor and acceptor fluorophore that are connected by a molecular spring (e.g. a spider silk protein). This allows for the FRET signal, which reports on the distance between the donor and acceptor, to be converted into the force acting on the spring (typically in the pN range). Such sensors are now well established for assessing the forces acting on E-cadherin, both at cell–cell junctions or when bound to substrate-immobilised E-cadherins (Borghi et al., 2012; Zhao et al., 2020).

Although FRET force sensor designs commonly use a spider silk protein as the molecular spring, newer variations of force sensors employ DNA structures. Molecular springs such as spider silk protein stretch continuously with the force acting on them, making them a powerful tool for reporting on force magnitudes acting on a group of ligands. They are, however, less suited for estimating the forces acting on single ligands. In contrast, dynamic DNA hairpin structures offer a different mode of action – they unfold reversibly upon application of a threshold force, leading to a rapid extension of the distance between donor and acceptor, similar to a digital response. DNA hairpins can be designed to have threshold unfolding forces with high accuracy and can cover a wider range of forces than are usually accessible with classical FRET sensor modules (Ma and Salaita, 2019). The combination of multiple FRET donor–acceptor pairs that are separated at different threshold forces also allows for a higher resolution of force measurement (Keshri et al., 2021). Some studies using FRET tension sensors are limited by their reliance on ratiometric methods to obtain a readout on the rate of FRET, which might not directly correlate to the average distance between donor and acceptor (Eder et al., 2017; VanBeek et al., 2007). Fluorescence lifetime imaging microscopy (FLIM) of the donor and acceptor (FRET-FLIM) is an alternative way to read out the average distance, offering the advantages that the fluorescence lifetime is largely independent of the probe concentration and that it requires readout of only a single fluorophore, which eases the complexity involved in imaging multiple probes (Keshri et al., 2021; Prechova et al., 2022).

Another interesting innovation in molecular tension sensors is the STReTCh tension detection module (Zhong et al., 2022), which employs the I10 domain of the muscle protein titin, known to undergo reversible unfolding upon a threshold force (Rief et al., 1997), linked to a SpyTag domain. Upon stretching, the SpyTag becomes accessible and can be bound by a fluorescently labelled SpyCatcher. This creates a fast ligand-binder system that is orthogonal to mammalian cell molecules, resulting in minimal cross-reactivity with other endogenous proteins. (Zakeri et al., 2012). Only molecules that experience forces above the unfolding threshold of titin I10 (1–2 pN) will be labelled, making STReTCh one of the most sensitive molecular tension sensors available and enabling easy readout in both cells *in vitro* and tissues.

Optogenetic approaches, referring to tools that control cellular functions using light, have also been employed to study AJ dynamics. E-cadherin trans-interactions can be locally and reversibly controlled using blue light with OptoE-cad (Nzigou Mombo et al., 2023). Key to this strategy is the LOV2 domain from *Avena sativa* phototropin 1 (AsLOV2, 404–542), which can undergo a reversible conformational change upon blue light illumination and has been widely used for optogenetic tools (Dagliyan et al., 2016). In OptoE-cad, the LOV2 domain is inserted between the first and second extracellular domains of E-cadherin, close to the Ca^2+^-binding sites. When LOV2 is in its folded state, Ca^2+^ can bind to OptoE-cad and E-cadherins on neighbouring cells can interact. Unfolding of LOV2 upon illumination with blue light results in the loss of Ca^2+^ binding and the unbinding of E-cadherins between cells. Another optogenetic strategy to disrupt AJs is to unlink the CCC from the underlying actin cytoskeleton. Photocleavable cadherin (PC-cadherin) and light-induced dissociation of adherens junctions (LInDA) are E-cadherin constructs that allow photocleavage of their intracellular domains, which associate with β-catenin and α-catenin, respectively, when exposed to blue light (Endo et al., 2019; Ollech et al., 2020). PC-cadherin employs the genetically encoded module PhoCL, which originated from the monomeric photoconvertible fluorescent protein mMaple and undergoes dissociation when stimulated with 405 nm light (Zhang et al., 2017). LInDa employs small photocleavable dimerisers that link the E-cadherin cytosolic tail with the transmembrane domain of E-cadherin, and stimulation with 350–405 nm light dissociates the complex and leads to the loss of AJs (Ballister et al., 2014). These tools have been used to study phenotypic changes, such as alterations in cell monolayer tension and cell surface area, that occur when the actin cytoskeleton is dissociated from the CCC, and allow for local disruption of AJs between cells and within tissues with fully formed cell–cell junctions. Beyond cadherin-specific optogenetic constructs, photoactivable RhoA and Rac1 offer the opportunity to alter actin cytoskeleton activity within cells (Cai et al., 2014; Cavanaugh et al., 2022). Together, these optogenetic tools are powerful approaches for changing the mechanical coupling between the components within AJs and the overall actin cortex activity in live cells. They offer high spatial and temporal control, thus allowing study of how these changes affect the interaction between cells and the integrity of tissues.

Functionalised substrates are widely used to study mechanical and organisational aspects in integrin-based cell adhesion but can also be employed for understanding E-cadherin-based cell adhesion. Soft, elastic substrates that are decorated with the recombinant and purified extracellular domain of E-cadherin are well suited to study cell contractility during adhesion as well as the role of substrate stiffness on actin organisation within the cell. The most prominent options for such substrates are polydimethylsiloxane (PDMS) elastomers or polyacrylamide (PAA) gels, whose stiffness or Young's modulus *E* (defined as the stress-to-strain ratio, or the force per unit area required to create a proportional deformation) can be controlled during their preparation. Although the stiffness of both materials can be tuned from a few hundred pascals (very soft) to over 100 kPa (stiff) in case of PAA (Mih et al., 2011) or even MPa for PDMS (Seghir and Arscott, 2015), they differ in their chemistry. PAA hydrogels are hydrophilic, making them biocompatible, while also being inherently non-adhesive to cells, allowing for attachment of specific adhesion molecules by covalently linking them to the hydrogel. Unlike PAA, PDMS elastomers are hydrophobic non-aqueous gels, which makes them a more suitable material for long-term experiments requiring a constant mechanical environment. This necessitates substrate treatments to prevent non-specific binding of proteins. Experiments using PDMS have, for example, shown that cells adhering to 0.3 kPa substrates exhibit fewer actin cables decorated with AJs compared to cells adhering to 2.4 kPa substrates (Eftekharjoo et al., 2022). Sub-micrometre-size deformable pillars made of PDMS decorated with E-cadherin have also been developed to study rigidity sensing between individual AJ complexes within a cell, showing that knockdown of α-catenin and vinculin leads to a decrease of AJ formation attempts (Yang et al., 2022).

Functionalised substrates are also relevant for studying the role of lateral mobility of cell adhesion molecules in AJ formation and cell cortex organisation. This can be achieved by using supported lipid bilayers (SLBs), usually formed onto glass substrates, to which the extracellular domain of E-cadherin or similar proteins can be tethered using functionalised lipids. The mobility of the attached proteins can be tuned by modulating the composition of the lipid bilayer. A big advantage of SLBs containing immobilised E-cadherin is that cells can reorganise E-cadherins along the SLB as occurs in a cell–cell adhesion. Using this approach, E-cadherin mobility has been shown to affect the maturation of cell adhesions, with low mobility fostering the formation of stable cell–SLB adhesion with clear differences in cortical actin flow and E-cadherin redistribution compared to adhesions of cells to highly mobile E-cadherins (Arslan et al., 2024; Biswas et al., 2015). These E-cadherin substrates allow for the observation of AJ formation and actin regulation in highly controlled physical environments. Additional tools such as micropatterning, when used in conjunction with E-cadherin substrates, can allow for further control of the physical environment.

The versatility of substrate functionalisation can be improved by adding spatial patterning. For the generation of patterns on a length scale of 10 µm and above, microcontact printing, where a PDMS stamp is used to deposit the molecule of choice onto a substrate, can be used. This approach has been used to shape cell doublets into simple geometries (e.g. squares, triangles and circles), showing that cell geometry affects the actin network and AJ organisation at the cell–cell interface (Sri-Ranjan et al., 2022). Micropatterning is typically a multi-step process involving the illumination of a substrate with a pattern of UV light, which usually limits the resolution of the pattern features to 0.2–1 μm. Most commonly, the substrate is first passivated (e.g. with a long-chained molecule like poly-ethylene glycol) and the passivation is then locally degraded upon UV light illumination, after which the functional molecule is then adsorbed onto these free sites, forming the micro-pattern (Azioune et al., 2010). An interesting application of this technique is the patterning of SLBs to produce heterogeneous surfaces with varying ligand mobility (Colin et al., 2023). Another powerful approach to modifying the mobility of ligands and lipids in SLBs is the use of chromium nanoimprint lithography (nanopatterning). This method produces nanoscale grid patterns (e.g. 0.5 to 2 µm squares, with 5 nm height in 100-nm-wide lines). These chromium grids allow the formation of a uniform SLB in which lipids and lipid-tethered ligands remain restricted within the small squares (Glazier and Salaita, 2017). This approach has been used to show that restricting E-cadherin mobility and preventing micrometre-scale clustering of E-cadherin reduces the proportion of α-catenin in an open conformation. However, once α-catenin undergoes the change to an open conformation, individual α-catenin–E-cadherin complexes are sustained even in the absence of force (Biswas et al., 2016).

The design and development of these new techniques and tools have enabled novel discoveries about AJ behaviour that were previously difficult to fathom. The biophysical methods highlighted here are, of course, only a subset of the many approaches being developed to manipulate and measure the mechanical properties of cells. These were chosen as examples of tools that can be combined to dissect the role of different mechanical factors in the formation and maintenance of AJs, not only at a molecular level but also in more complex and biologically relevant scenarios.

## Discussion

The fields of cell–cell adhesion and tissue morphogenesis provide wonderful examples of how the inclusion of biophysical perspectives has greatly advanced our understanding of fundamental biological processes. We have discussed here the molecular details of the CCC and actin contractility – which have now become extremely well established – along with a number of techniques well suited for the study of their function in cell culture systems. Although the role of dynamic maintenance and polymerisation of actin networks in maintaining junction stability is clear, several questions regarding the minutiae of their regulation remain open. This is likely due to the complicated nature of several converging pathways that are activated or inactivated, resulting in turnover processes, such as endocytosis and active remodelling of actin networks. The effects of AJ distribution and their associated actin architecture on junction stability and mechanics in live tissues still remain to be deciphered due to difficulties in imaging, creating genetic modifications and limitations in delivering local mechanical perturbations in animal models. The interplay between these different paths to AJ stability and how regulation of one could give rise to another aspect is still to be fully understood.

Finally, we draw attention to a few tangential points that, although not fully under the scope of this Review, no discussion of AJs would be complete without. We additionally highlight a few open questions that we find fascinating and hope to see answers to in the coming years. Cells within tissues are held together not only by AJs but also by the integrity of their basement membrane as well as by other types of junctions, such as desmosomes (Töpfer, 2023; Garcia et al., 2018). Furthermore, the actin cytoskeleton of adjacent cells is not only connected by AJs but also by tight junctions. Tight junctions, the most well-known constituents of which are occludins, claudins and zonula occludens (ZO) proteins, form at the apical extreme of epithelial tissues just above AJs. Whereas AJs form a thick band that provides strength to the cell–cell contact, tight junctions arrange in a thin band that is essential for maintaining the impermeability of the epithelial barrier. Thus, the formation of tight junctions is intrinsically linked to the maintenance of AJs both mechanically as well as through biochemical signalling pathways (Campbell et al., 2017).

Next, considering the distribution and balance of forces in a tissue, cells likely experience different forces at tricellular junctions compared to those at simple cell–cell contacts (Arnold et al., 2019). Given that AJs naturally are a point of mechanotransduction, this unsurprisingly leads to the recruitment of specific proteins, such as tricellulin and angulin (also known as MARVELD2 and ILDR1, respectively), that stabilise tricellular junctions. Developing tissues undergoing remodelling through processes such as convergent extension, in which cells change their neighbours in order to elongate the tissue in one direction, are characterised by transient tricellular junctions, whereas mature, stable tissues show fewer cell–cell junction transitions and more stable tricellular junctions (Higashi and Miller, 2017). During wound healing, dorsal closure during *Drosophila* development and similar morphological changes in other model organisms, a dense actin layer spanning multiple cells, commonly called a supracellular actin network, can be observed (Röper, 2013). However, this could be considered a misnomer, because if taken literally it suggests incorrectly that actin filaments are above or outside the cell. The supracellular actin network is more likely to be an outcome of accumulated TAFs that connect across membranes via AJs.

AJs are best studied in the context of the epithelium, but similar structures exist and are a subject of rising interest in other tissues, such as the endothelium. The main difference between epithelial and endothelial AJs lies in the type of cadherin that forms the AJs – endothelial cells express a homolog of E-cadherin called vascular endothelial (VE)-cadherin (also known as CDH5) (Lampugnani et al., 2018), whereas other tissues can express other cadherin variants such as N (neural)- and P (placenta)-cadherins, which form alternative CCCs (Franke et al., 2009; Hulpiau and van Roy, 2009; van Roy, 2014). Despite this naming of cadherin subtypes according to the tissues in which they were identified as the major variant, cells express a combination of these cadherins and can change their differential cadherin expression patterns during development or in diseases such as cancer (Halbleib and Nelson, 2006; Stemmler, 2008). The increasing availability of intravital imaging and higher order *in vitro* model systems, such as organoids (Corrò et al., 2020; Coste et al., 2020), will no doubt help shed more light onto AJ distribution and long-range actin architecture in live tissues.

The distinction between pAJs and linear AJs is so far only defined by their distribution along the cell–cell contact. Their molecular-level distinctions and what factors drive their formation is still to be understood. Interestingly, pAJs seem similar in structure to focal adhesions, which connect actin stress fibres to the extracellular matrix. The formation and regulation of focal adhesions is well studied and is dependent on various factors, including actin-generated forces, the activation of biochemical signalling pathways and lipid–protein interactions at the cell membrane (Romero et al., 2020; Kanchanawong and Calderwood, 2023). Given that several proteins found at focal adhesions are also recruited to pAJs, it will be interesting to see which of their associated signalling pathways are conserved and which ones vary between these two distinct structures. For example, is there a difference in CCC formation depending on the directionality of forces – i.e. forces parallel to the cell membrane, lateral forces acting on E-cadherins (e.g. due to membrane mobility and clustering of E-cadherins) and forces perpendicular to the cell membrane (e.g. by actomyosin contraction)?

The answers to these questions and many more exciting discoveries are expected as biophysical tools become increasingly commonplace. The genetic tools we have described will certainly be extended to study other molecular complexes involved in cell–cell adhesion, beyond those discussed here. Such developments will surely deepen our understanding of the link between the structure and dynamics of cell-cell junctions and their mechanical properties under different conditions.
